# Effects of free and encapsulated *Siah‐e‐Samarghandi* grape seed extract on the physicochemical, textural, microbial, and sensorial properties of UF‐Feta cheese

**DOI:** 10.1002/fsn3.3378

**Published:** 2023-05-04

**Authors:** Seyed Saeed Sekhavatizadeh, Nasim Abadariyan, Laya Ebrahimi, Mahboobeh Hasanzadeh

**Affiliations:** ^1^ Department of Food Science and Technology Fars Agricultural and Natural Resources Research and Education Center, AREEO Shiraz Iran; ^2^ Department of Food Science Kherad Institute of Higher Education Bushehr Iran; ^3^ Department of Food Hygiene and Public Health, School of Veterinary Medicine Shiraz University Shiraz Iran; ^4^ Department of Fisheries Academic Center for Education Culture and Research, ACECR Bushehr Iran

**Keywords:** emulsion, encapsulation, grape seed extract, polyphenol

## Abstract

The current study was conducted to elucidate the impact of grape seed extract (SE) and microencapsulated seed extract (MSE) addition to UF‐Feta cheese. The SE was encapsulated in maize starch, alginate, and canola oil using the emulsion technique. The SE and MSE characteristics were evaluated. The products were subjected to physicochemical (pH, titrable acidity, color, texture, and sensory properties), microbiological analysis (starter count), and lipid oxidation test (proxide, acid degree, and ansidine value) during 60 days of storage. The main phenol component in the SE was catechin (419.04 mg/L), gallic acid (319.63 mg/L), and chlorogenic acid (4.19 ± 0.002 mg/L). The antioxidant value was 157.80 mg/L. The MSE was elliptical in shape with a 24.29 μm diameter. The efficiency of microencapsulation was 53.86%. The addition of SE and MSE had no significant effect on pH and acidity, but lipolysis decreased based on acid degree value (0.7%; *p* > .05). The increasing trend of peroxide values was 172.54%, 145.68%, and 118.75% for C, SE, and MSE samples, respectively, and 35.68%, 32.28%, and 17.24% for the *P*‐anisidine values during the storage time. Therefore, fat oxidation was reduced in the supplemented cheese. Nevertheless, the supplemented cheese had limited color alterations. The MSE and SE did not affect the survival rates of the starter count. The SE and MSE had a less rigid structure. The hardness (2748.0 g) and chewiness (57.45 mJ) values in SE cheese had the greatest value among the samples. All sensory parameters were lowest in MSE cheese. In short, encapsulation showed suitable properties for SE to apply in UF‐Feta cheese.

## Graphical abstract

Emulsion is a suitable technique for encapsulation of grape seed extract. The grape seed extract and microencapsulated form did not affect the survival rate of cheese starter culture. The cheese containing microencapsulated grape seed extract had a good odor, texture, and overall acceptability on the 60th day of the storage time.

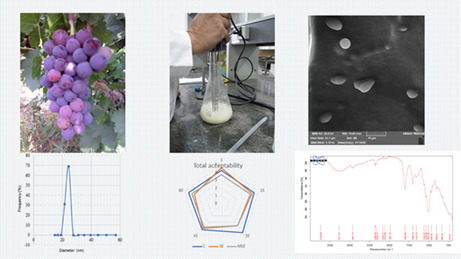

## 1 INTRODUCTION

Fermented dairy products, as a rich source of nutrients, are considered vulnerable foodstuffs which take part in the progress of spoilage and pathogenic microorganisms. The few suitable environments for the proliferation of microorganisms in the ripened cheese are moisture, temperature, and ripening time (Mareze et al., 2022). The oxidation susceptibility of lipid oxidation in various foods, including cheese, has been the subject of extensive research, although there are a limited number of reports focusing on protein oxidation (Govari et al., 2022). Synthetic preservatives currently being used in food systems have been reported to exhibit carcinogenic effects on humans. In recent decades, special attention has therefore been given to natural or nature‐friendly food preservatives, which are biodegradable with a favorable safety profile for humans and the environment (Singh et al., 2021). In recent years, consumer interest in functional foods, especially dairy products, has increased. However, the role of grape‐based by‐products is to diminish environmental pollution and also to develop the functional properties of dairy products (Kandylis et al., 2021). Grape as a flowering plant is a member of the family *Vitaceae*. The species of *Vitis vinifera* L. is an endemic Mediterranean plant. A large amount of marc consisting of seeds, peels, remaining portion of pulp, and stalks are produced following pressing grape bunches for different applications. The phenolic constituents of grape pomace including peels and seeds are mostly concentrated in the seeds of both red and white grape marc. The presence of phenolic compounds in the seed aqueous extract make it a valuable natural antimicrobial component to avoid food spoilage and foodborne infections (Maamoun, 2022).

Compared to pomace and skins, the seed extract displayed the highest 2,2‐diphenyl‐1‐picrylhydrazyl (DPPH; i.e., lowest EC50 values; Chikwanha et al., 2022).

Grape seed extract inhibits the formation of free radicals, chelate catalytic metals, and scavenges free radicals in biological systems. Furthermore, grape seed extract had suitable antioxidant activity in β‐carotene linoleate, linoleic acid peroxidation, DPPH, and phosphomolybdenum complex methods (Ma & Zhang, 2017; Razmaraii et al., 2017). The main chemical subclasses accumulated in grape seeds resulted from the polyphenols, the diverse group of flavan‐3‐ols, such as monomeric catechins and procyanidins (also known as condensed tannins). The grape seeds contain about 60 to 70% extractable phenolic compounds, and other chemical classes, including phenolic acids, stilbenes, flavonols, hydrolyzable tannins (including gallotannins and ellagitannins), and organic acids (Padilla‐González et al., 2022).

*Siah‐e‐Samarghandi* (*Vitis vinifera* L.) is one of the major grape cultivars grown in the north and northwest of Fars province, Iran. *Siah‐e‐Samarghandi* is a grainy, dark‐purple color with medium‐to‐late ripening time (Karami, 2011). Different biological activities including natural antioxidants, preservatives, and food fungicides are exhibited by consuming the grape seed extract to prevent food contamination by harmful microorganisms (Chen et al., 2020). Besides using the by‐product of grape seed extract in the wine and grape industry, it is also considered a GRAS (Generally Recognized as Safe) food additive. Bioactive compounds such as proanthocyanidins, epicatechin, catechin, and gallic acid are additionally present in the grape extract seed. Because of its abundant source, higher‐ranking health‐beneficial function, and antioxidant ability, grape seed extract has obtained great popularity and attention in recent years (Zhao et al., 2020). Many studies have recently focused on different properties of the bioactive compounds of grapes including antioxidant, antidiabetic, anticancer, antibacterial, antifungal, anti‐inflammatory, anti‐acne, anti‐aging, antihypertensive, protective effect, anti‐asthma, antiplatelet, anticataract, anti‐obesity, anticholinergic, anti‐sunburn, anti‐hyperpigmentation and wound‐healing (Parihar & Sharma, 2021).

Because of the natural appeal of the extracts of various herbs, a considerable reduction in using synthetic chemicals is predictable, especially in the supplemented dairy products. The Petit Suisse cheese manufactured with grape seed extract revealed a sensory acceptance rate of 73%. Moreover, the total phenolics and chemical antioxidant activity of Petit Suisse cheese were improved by using the combination of seed extract and skin (Deolindo et al., 2019).

Supplementation of grape seed extract into dairy products remarkably resulted in reducing syneresis from the interaction of phenol compounds with milk caseins to make stable complexes, which finally produce greater stability of the casein network. Several studies are recently focused on the addition of grape seed extract into numerous dairy products including cheese and yogurt (Brahmi et al., 2021; Deolindo et al., 2019; Han et al., 2019; Kavak & Akdenİz, 2019). Natural antioxidants are susceptible to chemical changes caused by factors such as pH, light, oxygen, temperature, and limiting their use in food. Therefore, adding natural antioxidants directly to food loses its antioxidant activity, especially during heating (Sharma et al., 2019). The stability to oxidation, controlled release, and thermal degradation, following encapsulation of polyphenols by different shell materials such as polysaccharides, proteins, and lipids also introduce new features, which are repeatedly reported. An emulsion is a suitable approach to encapsulating polyphenols (Cao et al., 2021).

Emulsification refers to the process of altering immiscible mixed liquids into an emulsion via homogenization. In recent years, numerous construction techniques have been successfully reported. However, a single emulsion (O/W or W/O) was considered a feasible method for the encapsulation of polyphenols (Wang et al., 2022).

Some limited research also suggested that microencapsulated grape seed extract (bead) may be incorporated into the yogurt as an additive or ingredient to introduce extra health benefits of products (Yadav et al., 2018). This is the first effort at the phenolic compounds and antioxidant properties of the aqueous extract in the seed of the *Siah‐e‐Samarghandi* grape cultivar, encapsulation of the seed extract using emulsion, and the addition of these encapsulated forms of cheese. The goal of this research is to evaluate the impact of MSE and the SE addition to UF‐Feta cheese and the determination of physicochemical and sensory properties.

## 2 MATERIALS AND METHODS

### 2.1 Collection and identification of the plant

The fresh *Siah‐e‐Samarghandi* grapes were collected from Fars province (south of Iran) on March 2021. The grape genus was recognized by a senior plant taxonomist at the Department of Botany, Fars Agricultural and Natural Resources Research and Education Center, Shiraz, Iran.

### 2.2 Chemicals and materials

Elx818 was applied to investigate the antioxidant activity. DPPH was obtained from the Sigma‐Aldrich company. Reference standard of 17 polyphenols (*p*‐comaric acid, trans‐ferulic acid, catechin, carvacrol, eugenol, chlorogenic acid, rosmarinic acid, quercetin, gallic acid, caffeic acid, ellagic acid, rutin, hesperetin, vanillin, hesperidin, coumarin, and sinapic acid) were also obtained from the Sigma‐Aldrich company for HPLC analysis. Canola oil was purchased from Aseel, United Foods Company, Al Quoz, UAE.

### 2.3 Preparation of methanolic extract

To form a fine powder, a grinder (Moulinex AR1100, Coulsdon, Surrey) was applied to ground dried seeds. The powder was used for methanolic extraction using the maceration technique by soaking the definite weight of powdered seed in methanol (1:10) for about 24 h and then through a 0.45‐μm Millipore filter. Then, it was injected into the HPLC for polyphenolic determination. The liquid residue was then concentrated by a rotary evaporator at 40°C for antioxidant activity analysis (Bahmanzadegan et al., 2019).

### 2.4 Determination of antioxidant activity (DPPH)

In order to evaluate the standard antioxidant activity of SE, the DPPH free radical scavenger assay was employed. Gallic acid was the standard compound for this test. In a modified assessment (Burits et al., 2001), 20 μL of 12.5–3200 μg/mL methanol extracts and gallic acid were added to 200 μL, 100 mM solution of DPPH radical in methanol, respectively, and solutions were placed at 27°C for 0.5 h. The microplate reader was used to measure DPPH radical inhibition at 515 nm. The Matlab software was applied for the calculation of IC50 of each sample (concentration in mg/L required to inhibit 50% DPPH radical formation). The methanolic solution extract excluding DPPH was used as the blank sample. The DPPH free radical scavenger method was employed to calculate the standard antioxidant activity of SE (Bahmanzadegan et al., 2019).

### 2.5 HPLC analysis of polyphenol

The modified procedure of Justesen et al. (1998) was used for the polyphenol extraction. To achieve the maximum sensitivity of the assay, gradient elution was selected. Different types of elution were prepared by changing the ratios of ingredients in the solvent. The gradient ranged from solvent A (formic acid 1% v/v in deionized water) to solvent B (extra pure methanol). The specifications of the solution involved predetermined ratios. The durations of HPLC operation were fixed accordingly. The ratios and durations methanol:formic acid 1% (10:90), at 0 min; methanol:formic acid 1% (25:75), at 10 min; methanol:formic acid 1% (60:40), at 20 min; methanol:formic acid 1% (70:30), at 30 min and finally, methanol:formic acid 1% (70:30), at 40 min. HPLC evaluation was executed on an Agilent 1200 series, prepared with a Zorbax Eclipse XDB‐C18 column (4.6 × 5 μmi. d.; × 150 mm film thickness, RP) and a photodiode array detector (PDA). Elution was monitored at 280 and 230 nm. The column temperature was 30°C. The injection volume was 20 μL. The standard solutions had a linear calibration curve with a good correlation (Mišan et al., 2011). Retention time and overlay curve were applied for the identification. It was clarified that all phenolic standards contributed with linear calibration curves through the concentration range (Table 1).

**TABLE 1:** Polyphenol content of the *Siah‐e‐Samarghandi* grape seed extract.

Polyphenol content	Concentration (mg/L)	Retention time (min)	Linear regression equation^a^	Correlation coefficient
Sinapic acid (mg/L)	ND	16.5	Y = 12.843x − 82.917	.999
Gallic acid (mg/L)	319.67 ± 0.03	3.3	Y = 1.2757x − 4.462	.994
Catechin (mg/L)	419.04 ± 0.07	8.3	Y = 9.2191x − 72.022	.996
Caffeic acid (mg/L)	ND	11.6	Y = 15.247x + 152.14	.997
Chlorogenic acid (mg/L)	4.19 ± 0.002	10.5	Y = 24.112x − 8.6696	.999
Quercetin (mg/L)	ND	21.6	Y = 14.927x + 72.349	.998
p‐Coumaric acid (mg/L)	ND	15.6	Y = 82.241x + 287.72	.999
Coumarin (mg/L)	ND	17.4	Y = 55.203x + 186.22	.999
Carvacrol (mg/L)	ND	28.4	Y = 10.675x − 12.921	.998
Vanilin (mg/L)	ND	13.5	Y = 42.74x + 59.464	.999
Trans‐ferulic acid (mg/L)	ND	16.3	Y = 30.718x − 214.48	.998
Hesperidin (mg/L)	ND	18.5	Y = 16.849x + 40.817	.997
Ellagic acid (mg/L)	ND	19.02	Y = 17.803x − 185.06	.992
Eugenol (mg/L)	ND	23.7	Y = 11.32x − 147.17	.993
Hesperetin (mg/L)	ND	22.4	Y = 30.574x − 141.76	.999
Rosmarinic acid (mg/L)	ND	19.2	Y = 24.232x − 101	.990

Abbreviation: ND, not detected.

^a^ Linear regression equation: Y = area; X = concentration.

### 2.6 Spectrophotometric determination of total phenolics

Folin–Ciocalteu reagents were used to spectrophotometrically measure the total phenol. The following equation was used to calculate the actual amount of gallic acid (c = 1.885 × A ± 2.81, R2 = .9953). Finally, four calibration points within the range of 6.25–50 mg/mL of gallic acid in the reaction mixture were plotted (Mišan et al., 2011).

### 2.7 Spectrophotometric determination of total flavonoid

The aluminum chloride colorimetric method was altered from the manual mentioned by Chang et al. (2002). Quercetin was used to construct the calibration curve. Ten milligrams of quercetin were dissolved in 80% ethanol and then diluted to 6.25, 12.5, 25, 50, 80, and 100 mg/L. The standard solutions (0.5 mL) were diluted and separately mixed with 0.1 mL of 10% aluminum chloride, 0.1 mL of 1 M potassium acetate, 1.5 mL of 95% ethanol, and 2.8 mL of distilled water. After incubating at 27°C for 0.5 h, the Shimadzu UV‐160A spectrophotometer was used to assess at 415 nm. The optical density of the reaction mixture was assessed. The blank sample consisted of an equal amount of 10% aluminum chloride that was replaced with an equal amount of distilled water. Similarly, the 15 flavonoid standard solutions (100 ppm) or 0.5 mL of ethanol extracts were reacted with aluminum chloride for assessing flavonoid content as stated above.

### 2.8 Microencapsulation of extract

A modified technique by Sultana et al. (2000) was used for microencapsulation. All glassware and solutions used in this protocol were sterilized at 121°C for 15 min. A 2% alginate mixture was prepared to contain 2% Hi‐maize resistant starch (Starch Australia Ltd.). The mixture was dropped into the canola oil, containing Tween 80 (0.02%). After dropping, the mixture was stirred vigorously till it was fully emulsified to the creamy form. A solution of 0.1 M calcium chloride was then added fast along the side of the beaker, and the phase separation of oil/water emulsion took place. To separate and settle the emulsion at the bottom of the calcium chloride layer, the mixture was allowed to stand for 30 min for the calcium–alginate beads. The oil layer was drained and the beads were collected by slow centrifugation (3503 *g*, 15 min), washed once with 0.9% saline containing 5% glycerol, and stored at 48°C. Bead size separation was performed using 500 and 150 mm steel screens.

### 2.9 Particle size, morphology, and microencapsulation efficiency

The aspect ratio of 20 beads has been analyzed using a digital microscope and Micromeasure software version 1.07. Equation 1 was employed to measure the aspect ratio (Wang et al., 2015).

(1) $$\text{Aspect}\;\text{ratio}=\text{Large}\;{\text{diameter}}_{\mathrm{mm}}/\text{small}\;{\text{diameter}}_{\mathrm{mm}}$$

A dynamic light scattering (DLS) device (90 Plus, Brookhaven Instruments Corp.) was used to measure the particle size distribution. Analyses have been done at a scattering angle of 90 at 25°C (Hosseini et al., 2013).

The establishment of the phenolic compounds was employed to verify the efficiency of encapsulation. The 800 mg of microencapsulated powder was accurately weighed, added to 4 mL of methanol (as a solvent), and gently shaken using a vortex for 2 min at room temperature. The tube was then centrifuged (IEC Centra3M Centrifuge) at 81*g* for 5 min. The Folin–Ciocalteu colorimetric method was used to measure the phenolic compounds in the slurry which were eventually called microencapsulated polyphenols (P encap; mgGA/gDE). The total polyphenol content of dried CE was assessed based on the Folin–Ciocalteu colorimetric method (P total; mgGA/gDE). To calculate the efficiency of microencapsulation, the following equation was then applied (Eencap; %, Ganje et al., 2016):

(2) $$\text{Eencap}\;\left(\%\right)=P\;{\text{encap}}_{\left(\text{mgGA}/\mathrm{gDE}\right)}/P\;{\text{total}}_{\left(\text{mgGA}/\mathrm{gDE}\right)}.$$

### 2.10 Fourier transform infrared spectroscopy (FTIR) of beads

To analyze the functional groups and provide information on the structural properties of the samples, FTIR spectroscopy was implemented by a Tensor II FTIR spectrometer (Bruker). Wavelengths of all spectra were recorded at 4000–400 cm^−1^ (Elnaz & Saeed, 2020).

### 2.11 Cheese samples preparation

Cheese samples were produced at Pegah Dairy Co. The bactofugation technique was used to physically remove somatic cells, spores, and bacteria from the milk. The milk was then pasteurized (at 76°C for 5 s), ultrafiltered, and homogenized. The milk (5.4 kg) to 1.0 kg ratio of the retentate was added to stable the milk tank. Ten grams of starter were then added for every ton of Feta cheese. The milk in the filler was set at pH 6.2 and rennet was added to cheese vat water (2 g per 100 kg of holding solution).

According to the outcomes of an organoleptic test, SE (5% w/w) was added to the milk. The ranking test was performed by 50 non‐educated panelists of both genders, ranging from 20 to 45 years of age, who were pre‐selected according to the expression of interest of the panelists. Various concentrations of SE (1%, 3%, 5%, 7%) were used to assess the flavor preference of cheese. Approximately, 20 g of each sample was served by every panelist randomly encoded with a three‐digit number. Based on their overall impression, the preference ranking test, subjects were asked to select the most and the least preferred samples. Finally, the total priorities were calculated. In order to apply an equal quantity of extract to each SE and MSE cheese, concentrations of approximately 10% (w/w) and 5% (w/w) for SE and MSE were added to each sample, respectively. Samples deprived of SE and MSE were considered as control (C). The retentate had to be converted to its pre‐cheese state and remain in the coagulation tunnel at 37°C for 30 min. Before sealing the aluminum foil, the cheese parchment paper was treated with 2% (w/w) salt. The cheese samples were placed in a refrigerator at a temperature of 4 ± 1°C and the cheese was left in place for 60 days, with the potential to support aging during the pre‐aging period (37°C). However, this phenomenon occurred after the pH drop of the cheese to 5.0 during storage. The organoleptic and structural properties of the samples were eventually analyzed during storage (Karami et al., 2009).

### 2.12 Proximate, pH, and acidity value of cheese

The moisture content was measured after drying at 103°C until a certain weight was reached. Proteins were measured according to the Kjeldahl method (N × 6.25) and crude fat was extracted with petroleum ether using a Soxhlet extractor (gravimetric method; Balogun et al., 2016). The pH values of the experimental groups (SE and MSE cheese) were recorded using a pH meter (Greisinger Electronic). The Dornic degree (National Standard of Iran, 2852) was then used to show the titrable acidity of the sample (Karimi et al., 2021).

### 2.13 Acid degree value (ADV)

The ADV of the samples was determined as described by Sulieman et al. (2013). Five grams of cheese samples weighed and added to 25 mL alcohol (ethanol), 25 mL diethyl ether, and 1 mL of phenolphthalein solution (1% w/v). the mixture was carefully neutralized with 0.1 mol/L sodium hydroxide and shaking constantly to obtain a pink color. The following equation was then used to calculate the acid degree values:

(3) $$\text{Acid}\;\text{degree}\;\text{value}=5.61\times {\text{titration}}_{\left(\mathrm{mL}\right)}/{\left(\text{sample}\;\text{used}\right)}_{\left(g\right)}.$$

### 2.14 Color

Cheese color was measured using a Chroma‐meter CR‐400 (Konica‐Minolta). The L* value is an indicator of lightness (black‐to‐white lightness). The a* values indicate green (−) and red (±), and b* indicates blue (−) and yellow (±).

### 2.15 Texture

The Texture profile analysis was employed to measure the texture of samples using the texture analyzer CT3, (Brookfield Engineering Laboratories, Inc.). The TPA of cheese samples was recorded on the first and the 60th days of storage. A sample (height 10 ± 0.5 mm, diameter 20 ± 0.5 mm) was taken from the center of the cheese. Next, the cylindrical cheese was covered with a stretch film and brought to room temperature of 20 ± 1°C. The following analysis conditions were as follows: TA11/1000 aluminum cylinder probe (25.4 mm in diameter), compression 20% of the initial height, test speed 1 mm/s, penetration rate 2 mm/s, pretest speed 2 mm/s, and retention time 5 s. The textural parameters including gumminess (g), hardness (g), chewiness springiness (mm), and cohesiveness were obtained from the device (Oluk et al., 2014).

### 2.16 Sensory analysis

The sensory analysis of C, SE, and MSE cheeses was evaluated by 45 educated panelists. They were divided into two age groups: 18–24 years and 24–51 years (a total of 42% males and 58% females). The 5‐point hedonic scale was applied to the cheese samples. The flavor, odor, color, texture, and overall acceptability were assessed by panelists. A scale of 5 indicated “like extremely” and 1 “dislike extremely” in comparison with a C cheese. The samples were evaluated by the panelists every 15 days during the storage period (Dokoohaki et al., 2019).

### 2.17 Peroxide value (PV)

Peroxide value was analyzed using Siddique and Park (2018) method. Five grams of the previously extracted fat sample were weighed into a 250 mL Erlenmeyer flask. Thirty milliliter of acetic acid–chloroform solution (3:2 v/v) was added to each sample, and the contents were carefully swirled until properly dissolved. A 0.5 mL saturated potassium iodide was added to each sample and shaken for 1 min. Thirty milliliter of distilled water was added to each sample, and the sample was shaken vigorously. One milliliter of starch solution (1%) was added to each sample. The sample flask was titrated with 0.01 N sodium thiosulfate until the bottom layer appeared milky, which indicated the endpoint of the assay. Equation 4 was used to calculate the peroxide value (Siddique & Park, 2018):
(4)

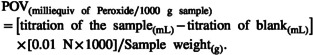




### 2.18 *p*‐anisidine value (AnV)

AnV is an indicator of the degree of lipid oxidation, especially stable secondary products resulting from the oxidation of lipids in foods. Measurements were performed using a DR5000 UV–Vis spectrophotometer (Hach Lange) and isooctane as a blank. AnV was determined according to Equation 5:

(5) $$\mathrm{AnV}=25\times \left(1.2\mathrm{As}-\mathrm{Ab}\right)/m$$

As, the absorbance of the sample; Ab, the absorbance of the blank; and m, the mass of the sample (g) (Ghendov‐Moşanu et al., 2020).

### 2.19 Starter viability

A starter viability assessment was performed on C, SE cheese samples, at 15‐day intervals during 60‐day storage. Sterile trisodium citrate (225 mL; 2% w/v) was used to dilute the cheese samples (25 g) at 40°C. Samples were infused in a Stomacher bag at high speed for 4 min with a Bagmixer 400 range (Interscience). This was done to obtain a slurry for initial dilution, but other serial dilutions were done using 0.15% (w/v) aqueous peptone solution. Appropriate dilutions were pour‐plated. *S. thermophilus* and *Lactococci* were examined in terms of their viability. M17 agar plates received 5 g 100 m/L sterile lactose before being placed in an incubator for 48 h at (30 ± 1)°C and 24 h at (43 ± 1)°C as treatments for the *Lactococcus lactis* and *S. thermophilus*, respectively, in anaerobic conditions (Katalinic et al., 2013; Ruggirello et al., 2014).

### 2.20 Statistical analysis

SPSS version 21.0 (IBM Corp.) was used to analyze the experimental data. ANOVA worked at (*p* ≤ .05) and Duncan's multirange test helped to perform a meaningful comparison. All experiments were performed three times, except in exceptional cases, which are otherwise stated in the text. All graphs are Microsoft Excel Ver. Created in. 16 (Microsoft).

## 3 RESULTS AND DISCUSSION

### 3.1 Polyphenol and total phenolic content

HPLC–UV analyses of phenolic compounds extracted from SE were carried out by the method validated on the standard phenolic compounds and expressed as mg L^−1^. A total of 17 investigated polyphenols are listed in Table 1. The main phenol component in the *Siah‐e‐Samarghandi* grape cultivar seed was catechin (419.04 ± 0.07 mg/L), gallic acid (319.63 ± 0.03 mg/L), and chlorogenic acid (4.19 ± 0.002 mg/L), respectively. Thymol, ellagic acid, sinapic acid, caffeic acid, eugenol, p‐comaric acid, rutin, carvacrol, quercetin, rosmarinic acid, hesperidin, trans‐ferulic acid, hesperetin, and coumarin were not detected in *Siah‐e‐Samarghandi* grape cultivar seed extract. Maamoun (2022) reported that seed flours also contained numerous components including gallic acid, ferulic, caffeic, catechin, epicatechin, epicatechin gallate, procyanidin B1, and procyanidin B2. In the current study, the total phenolic content was 273.89 ± 4.01 mg/g. In a research, total phenolic content ranged between 6711 and 8818 mg GA/g methanol extract (Maamoun, 2022). Moreover, Baydar et al. (2006) reported that the contents of total phenolic compounds of the grape seed extracts were (589.09, 506.60, and 549.54 mg/g) for Hasandede, Emir, and Kalecik Karasi cultivars, respectively. Alterations in the values likely resulted from different plant parts from which the raw plant material was taken (Borhanpour et al., 2021).

### 3.2 Antioxidant activity and flavonoid

The antioxidant activity of *the Siah‐e‐Samarghandi* grape seed was 157. 80 ± 5. 40 mg GAE/L which was greater than IC50 (142 ± 24 mg GAE/L) reported by Katalinic et al., 2013. The amount of phenolic compound and antioxidant activity in fruits of grape cultivars varied in different growing conditions (Salehi et al., 2013). The crucial role of procyanidin B1 as the most important radical scavenger in grape seed extracts was approved (Maamoun, 2022). In our studies, the total bioflavonoid content of grape seeds was found to be (5.25 ± 0.08 mg/g) quercetin equivalent. In a similar report, the total bioflavonoid was 6.892 mg/g (Al‐Habib et al., 2010). Grape seed extract corresponded to a compound with a flavan‐3‐ol structure, principally composed of procyanidins (99.45%), with only small quantities of catechin (0.32%) and epicatechin (0.23%; Martinez et al., 2005).

### 3.3 Particle size, the morphology of beads, and the efficiency of microencapsulation

**FIGURE 1:**
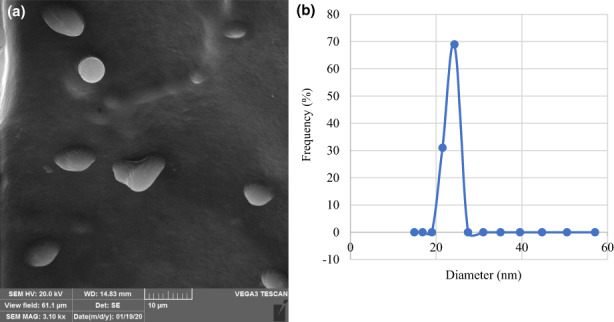
Scan electron microscopy (a) and droplet size of microencapsulated seed extract (MSE) of the *Siah‐e‐Samarghandi* grape seed extract (b).

All of the beads had an elliptical shape with a convex center (Figure 1a). The average particle size of the beads was 24.29 μm (Figure 1b). This result was not in agreement with those of the previous report by Matos et al. (2013) who reported that starch particles stabilized emulsion droplets in the range of 23–43 μm with an average diameter of 33 ± 7 μm. The particle size depended on the composition of the emulsion, and the size of the spherical particles ranged from 1 to 80 μm in diameter (Matsumoto et al., 2003). The interaction between amylose and lipids has been previously described by several authors, elsewhere. For example, the formation of complex particles with palmitic acid upon released forms of starch granules was reported (Meng et al., 2014). In this interaction, no starch emulsion presented more aggregation of oil droplets. However, in the emulsion‐containing starch, oil droplets were more dispersed, as observed in (Figure 1b; Feltre et al., 2020).

In this study, the efficiency of microencapsulation was 53.86%. In contrast, Carneiro et al. (2013) reported values of 62.3%–95.7% for encapsulation efficacy. The composition of the wall material used for encapsulation was the main reason for this variation, which greatly affects encapsulation efficiency.

### 3.4 FTIR of beads

**FIGURE 2:**
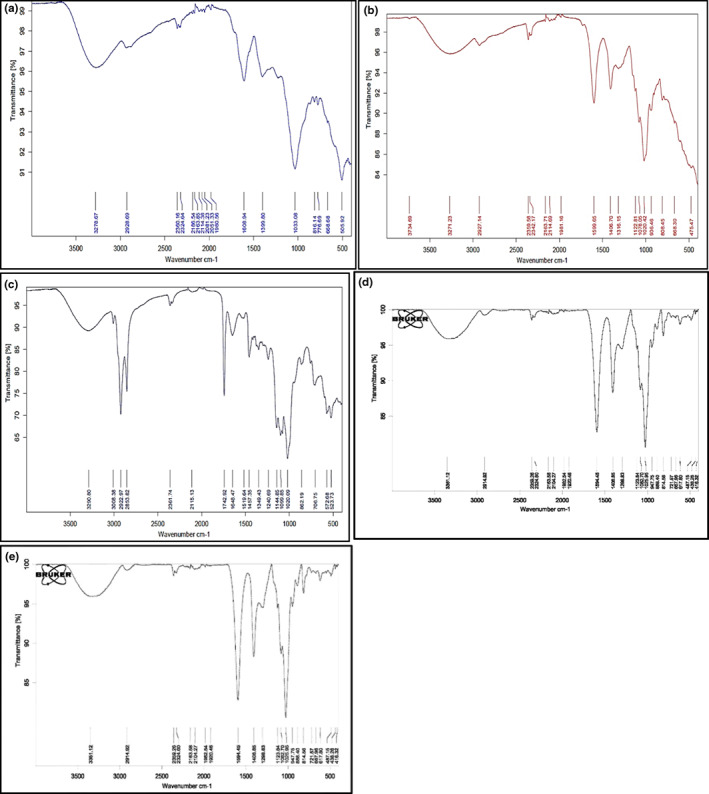
FTIR spectrums of the seed extract (SE) (a); seed extract containing Hi‐maize resistant starch, sodium alginate, and Tween 80 (b); seed extract containing Hi‐maize resistant starch, sodium alginate, Tween 80, and calcium chloride (c); sodium alginate (d); and maize starch (e).

The FTIR spectra of beads and microencapsulation components are presented in Figure 2a–e. The FTIR spectral analysis of all samples found bands of 3400 and 3427 cm^−1^ assigned to the vibration of OH in sugar units. The stretching hydrogen bands in C–H were indicated by bands ranging from 3200 to 2800 cm^−1^ (2922, 3008, 2927, 2928, 2853, and 2922 cm^−1^), and broadband at 1742 cm^−1^ exhibited the C=O stretching of the ester carbonyl functional group (Vlachos et al., 2006). This region is related to the triglyceride absorption bands in canola oil (Ozulku et al., 2017). This shows the intramolecular interaction between C=C and the hydrocarbon chains of unsaturated fatty acid segments such as C18: 1, C18: 2, and C18: 3 in canola oil (Soltaninejad & Sekhavatizadeh, 2019). As well, the other characteristic bands, such as 1457 cm^–1^ (bending vibration of C2H3 aliphatic groups), 1020, 1099, 1144, and 1240 cm^–1^ (stretching vibration of the C–O ester groups), were observed (Figure 2c). They agree with the results of Waterhouse et al. (2014). The last fingerprint region of FTIR spectra between 706 and 862 cm^–1^ wavelength frequency was ascribed to the CH2 rocking vibration and the out‐of‐plane vibration of cis‐disubstituted olefins (Ozulku et al., 2017). As shown in Figure 2c, there are two peaks in the absorption spectrum at 2853 and 2922 cm^−1^, which represent the C–H functional group (Munajad & Subroto, 2017). In all FTIR spectra, the bands ranging from 2000 to 2500 cm^−1^ are observed, which are related to the triple bands C≡N and C≡C (Nandiyanto et al., 2019). The two symmetric and asymmetric stretch peaks at 1594 and 1406 cm^–1^ can be associated with the functional group of O=C–O– stretching in the sodium alginate (Figure 2d). However, the symmetric one revealed a large shift toward high wavenumbers from 1594 to 1648 cm^–1^. The formation of a specific ion‐binding peak around the carbonyl group designed a new environment as the calcium ions were replaced by sodium ions in the alginate blocks (Anbinder et al., 2011). The characteristic bands of typical polyphenols produce the spectrum of the seed extract (Figure 2a). A broadband of around 3271 cm^–1^ allocated to the stretching modes of O–H groups and a shoulder at 2927 cm^–1^ that resembles the hydroxyl groups involved in an intermolecular hydrogen bond were observed. Besides, special peaks at 1599 cm^–1^ are attributed to the C=C vibration of an aromatic ring, in addition to the C–O stretching band at 1316 cm^–1^. Other hydroxyl‐related bands were observed at 1122 and 1078 cm^−1^. Upon forming the capsules of calcium alginate, the intensity of this band grew, following the stretching of the polyphenols band at 3271 cm^–1^ shifted to 3290 cm^–1^ (Anbinder et al., 2011). The strong characteristic peak at around 3315 cm^−1^ represents glucose O–H stretching vibration found in Hi‐maize starch (Figure 2e). The antisymmetric stretching vibration of CH2 was formed at the peak at 2928 cm^−1^, and that at 1638 cm^−1^ was the amorphous region vibration of starch (Mala & Anal, 2021). As shown in Figure 2b,c, no novel chemical bond and the functional group were observed in the FTIR spectra of normal maize starch complexed with the seed extract (polyphenol) compared to normal maize starch, which suggests that there is probably no covalent interaction between starch and polyphenols. Maize starch interacts with polyphenols via non‐covalent interactions such as hydrogen bonds and hydrophobic interactions as proposed in previous research (He et al., 2021).

### 3.5 Proximate value, pH, and acidity of the cheese

**FIGURE 3:**
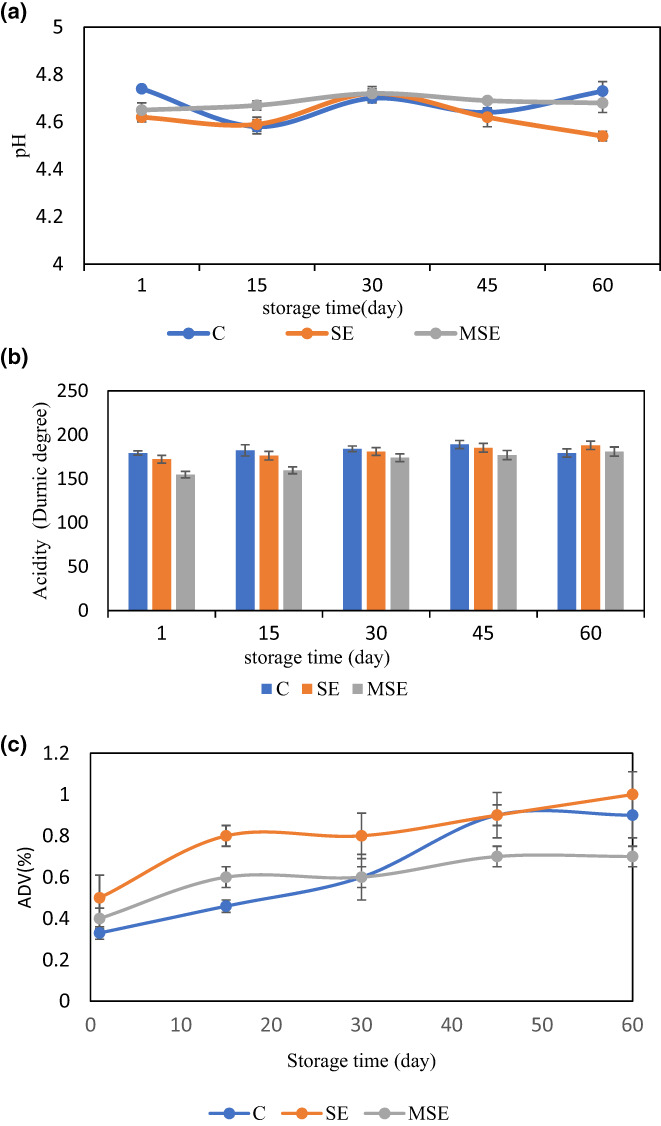
Chemical parameters of cheese samples supplemented with microencapsulated seed extract (MSE), seed extract (SE) of the *Siah‐e‐Samarghandi* grape, and control (C) cheese during the storage time. (a) pH, (b) Titrable acidity, and (c) Acid degree value (ADV); *Mean ± SD (*n* = 3).

Table 2 shows the changes in fat, protein, and water content of UF‐Feta cheese during aging. In terms of dry matter, in each sample, there was a significant (*p* < .05) difference between day 1 and day 60 of storage. There was also a significant increase in the dry matter of all cheeses in response to the loss of moisture. The content of protein in all treatments increased on the 60th day. These results were similar to those of Nasiri et al. (2020) who reported an increase in total protein content at the end of the storage period. The MSE cheese had the highest protein content, which reached from (14.05 ± 0.109% w/w) to (14.69 ± 0.90% w/w) during ripening. The content of fat in all treatments decreased during the storage time. Similar results were reported concerning fat quantity in UF‐Feta‐type cheese during storage time (Karami et al., 2008; Yazdanpanah et al., 2018). The results obtained in this study showed that the addition of SE and MSE to Feta cheese had no significant (*p* > .05) effect on pH changes during storage but increased acidity value (Figure 3a,b). These results reflect those of Kim et al. (2017) who reported that the pH values of the Gouda cheese supplemented with pepper extract beads were not significantly different from the control cheese at each time point. However, the pH of each sample did not significantly change during the aging period.

**TABLE 2:** Proximate value in cheese samples during storage time.

Parameters	Cheese sample	Storage time	
		1	60
Protein (w/w)	C	13.20 ± 0.54^aA^	14.12 ± 0.62^bA^
	SE	12.85 ± 0.74^aA^	13.50 ± 0.57^bA^
	MSE	14.05 ± 0.109^aA^	14.69 ± 0.90^aA^
Dry matter (w/w)	C	44.06 ± 0.17^aA^	44.47 ± 0.39^aA^
	SE	41.92 ± 0.28^aC^	42.25 ± 0.31^aB^
	MSE	43.41 ± 0.16^aB^	43.98 ± 0.61^aA^
Fat in dry matter (w/w)	C	47.50 ± 0.53^aA^	47.28 ± 0.75^aA^
	SE	47.60 ± 0.89^aA^	47.41 ± 0.46^aA^
	MSE	48.64 ± 0.57^aA^	48.01 ± 0.64^aA^

*Note*: Microencapsulated seed extract (MSE), seed extract (SE) of the *Siah‐e‐Samarghandi* grape, and control cheese (C). All the results are the means ± standard deviations of triplicate determinations. Data within the different letters (A–C) in column and (a–b) in a row are significantly different (*p* ≤ .05).

### 3.6 Acid degree value (ADV)

Acid degree value (ADV) was standing for the level of free fatty acids in the samples (Figure 3c). In the terms of ADV value, SE and MSE cheese samples had lower ADV values than the control at the end of the storage time. ADV value increased during the storage time in all samples. As a result of spontaneous lipolysis during cold storage. It may be related to an increase in the total aerobic count of cold‐stored cheese (Coskun & Ondul, 2004). This view is supported by Khan et al. (2018), who reported this process in the Guda cheese enriched with mango kernel oil (*Mangifera indica* L.). Folllowing 90 days of storage, the total free fatty acids was increased in all experimental groups. Bacterial lipases and moisture content played major roles in the generation of free fatty acids.

### 3.7 Color analysis

Results of color indices (a*, b*, L*) in the cheese during storage at 4°C have been presented in Table 3. Regarding the L* values which indicate lightness, no significant affection was shown (*p* > .05) among the cheese samples. Limited color alterations were due to colorless grape derivatives like grape seed extracts or white grape varieties (Kandylis et al., 2021). However, during storage time, the L* parameter of MSE samples was increased at the end of the storage period (45–60 days), although no difference was seen in the other samples during the storage time. The a* and b* values were equal among the samples but b* increased during the storage time. Cenobio‐Galindo et al. (2019) reported that the parameter b* was increased in all yogurts containing multiple emulsions with extracts of cactus pear and during shelf life.

**TABLE 3:** Color parameters of cheese samples during the storage time.

Parameters	Day	C	SE	MSE
L*	1	54.44 ± 1.45^aB^	60.89 ± 2.42^aA^	57.33 ± 1.93^aAB^
	15	53.0 ± 0.81^aB^	57.33 ± 1.22^aA^	52.78 ± 2.44^aB^
	30	58.0 ± 1. 70^aAB^	55.89 ± 1.87^abA^	51.89 ± 1.31^bB^
	45	62.78 ± 2.57^aA^	59.67 ± 2.75^aA^	62.11 ± 2.47^aA^
	60	54.44 ± 2.33^aB^	62.89 ± 2.49^aA^	60.67 ± 3.38^aA^
a*	1	−2.33 ± 0.52^aA^	−2.22 ± 0.57^aA^	−0.67 ± 0.4^aA^
	15	−2.22 ± 0.57^aA^	−2.0 ± 0.5^aA^	−2.22 ± 0.64^aA^
	30	−2.0 ± 0.57^aA^	−1.89 ± 0.53^aA^	−1.44 ± 0.85^aA^
	45	−1.44 ± 0.76^aA^	−2.44 ± 0.55^aA^	−2.11 ± 0.63^aA^
	60	−1.67 ± 0.55^aA^	−2.0 ± 0.44^aA^	−2.33 ± 0.66^aA^
b*	1	15.56 ± 0.37^aC^	15.0 ± 0.76^aB^	16.33 ± 0.98^aA^
	15	16.11 ± 0.92^aC^	15.0 ± 1.0^aB^	15.78 ± 2.29^aA^
	30	17.11 ± 0.96^aBC^	17.56 ± 0.89^aAB^	17.33 ± 0.86^aA^
	45	19.44 ± 1.26^aAB^	17.44 ± 0.62^aAB^	16.44 ± 0.85^aA^
	60	20.44 ± 1.48^aA^	18.22 ± 0.86^aA^	17.11 ± 1.11^aA^

*Note*: Microencapsulated seed extract (MSE), seed extract (SE) of the *Siah‐e‐Samarghandi* grape, and control cheese ©; L* is the luminance or lightness component, a* (from green to red), and b* (from blue to yellow). All the results were the means ± standard deviations of triplicate determinations. Data within the different letters (A–C) in column and (a–b) in a row are significantly different (*p* ≤ .05).

### 3.8 Texture

In this study, texture profile analysis correlates well with sensory parameters, a beneficial index of the textural excellence of a cheese product. In the current study, SE and MSE were two factors that affected on textural parameters of cheese. The SE and MSE had a less rigid structure. Texture profile analyses of cheese samples are given in Table 4. Hardness values were higher in C and SE cheeses than that in MSE on the first day of the storage time. Moreover, hardness increased during storage time in each sample. The decrease in moisture content may be responsible for the increase in hardness due to the greater hydration and consequent weakening of the casein network during storage time (Pereira et al., 2001). The decrease in pH value may be another factor in the observed increase in hardness. The interactions between polyphenols and macromolecules have been widely reported, elsewhere. Such interaction was mostly investigated between proanthocyanidins and proteins, from which proline‐rich amino acids revealed the greatest affinity. The association was also shown between phenolics with polysaccharides, metallic ions, and lipids. This occurred through non‐covalent (i.e., van der Waals forces, hydrogen, and hydrophobic and ionic bonding) and covalent bonds (including the formation of carbocations) binding property of phenolics with macromolecules (Chikwanha et al., 2022).

**TABLE 4:** Texture analysis in cheese samples in the storage time.

Texture parameters	Cheese type	Storage time	
		1	60
Hardness (g)	C	815.75 ± 4.6^bA^	3309.0 ± 333.7^aB^
	SE	625.5 ± 4.95^bB^	4497.0 ± 176.07^aA^
	MSE	501.0 ± 1.41^bC^	2748.0 ± 72.12^aB^
Cohesiveness	C	0.83 ± 0.11^aA^	0.54 ± 0.08^aA^
	SE	0.45 ± 0.03^aB^	0.57 ± 0.1^aA^
	MSE	0.66 ± 0.14^aA^	0.51 ± 0.03^aA^
Adhesiveness (mJ)	C	0.66 ± 0.1^aA^	0.73 ± 0.26^aA^
	SE	0.54 ± 0.11^aA^	0.7 ± 0.08^aA^
	MSE	0.66 ± 0.05^aA^	0.83 ± 0.56^aA^
Chewiness (mJ)	C	97.24 ± 3.34^aA^	74.04 ± 16.65^aB^
	SE	45.90 ± 3.82^bB^	107.49 ± 4.82^aA^
	MSE	39.98 ± 2.09^aB^	57.45 ± 1.99^aB^
Springiness (mm)	C	1.57 ± 0.47^bA^	4.18 ± 0.12^aA^
	SE	1.23 ± 0.33^bA^	4.44 ± 0.28^aA^
	MSE	1.22 ± 0.23^aA^	4.16 ± 0.2^aA^

*Note*: Microencapsulated seed extract (MSE), seed extract (SE) of the *Siah‐e‐Samarghandi* grape, and control cheese (C). All the results were the means ± standard deviations of triplicate determinations. Data within the different letters (A–C) in column and (a–b) in a row are significantly different (*p* ≤ .05).

The results also showed that in the SE cheese, cohesiveness, springiness, and adhesiveness values are equally compared to the (C). But chewiness in the SE cheese was greater than the others following the 60th of storage. The results showed that the chewiness, adhesiveness, and cohesiveness values of all cheeses were constant during storage, but springiness increased. The chewiness was a secondary texture parameter of cheese. The number of chews required to be ready to swallow the sample (Gwartney et al., 2004). Proper chewing ability guarantees a rich mouthfeel and boosts the pleasure of tasting cheese. Higher water and fat content will reduce the hardness of the cheese. In contrast, the higher the contents of dry matter and protein, the harder the cheese (Zheng et al., 2016). The results of this study were similar to those of Paximada et al. (2021). They made fortified cheese containing double emulsion. Statistically, the results demonstrated that the cheese‐containing emulsion has the lowest hardness (20 N). Springiness is a term to describe the height of cheese recovers after the first and before the second compressions (Sahan et al., 2008). Low‐fat cheese is generally recognized as springy. The strong structure of cheese is associated with scattered fat globules between the protein chains. As fat increases, the protein matrix becomes thinner, and more fat globules are distributed, which makes the structure less compact (Johnson et al., 2009; Ustunol et al., 1995). Therefore, with MSE cheese, the springiness decreases as the fat content increases (Tables 2 and 4). For example, the springiness of MSE cheese decreases with increasing fat content. In research by Choi et al. (2017), similar to current studies, springiness was lower than controls for all cheeses containing W1/O/ W2 double‐emulsion beads. The amount that simulates the force of the inner bond is defined as the cohesive force (no dimension; Sahan et al., 2008). In similar research, cohesiveness and adhesiveness are equal between liposomal encapsulated saffron extract and control Ricotta cheese (Siyar et al., 2022). This result is in agreement with our observation. A decreased level of cohesiveness was observed during maturation, this finding was supported by Fox et al. (2017), who found that this coincides with decreasing fat content, and a decline in cohesiveness was seen. Van Hekken et al. (2007) showed lower levels of cohesiveness of Chihuahua cheese, which ranged from 0.19 to 0.31, lower than those obtained in this research (0.51 ± 0.03–0.66 ± 0.14), however, it was close to the findings of Pimentel‐González et al. (2015), who reported Chihuahua cheese with antioxidants in multiple emulsions.

### 3.9 Sensory analysis

**FIGURE 4:**
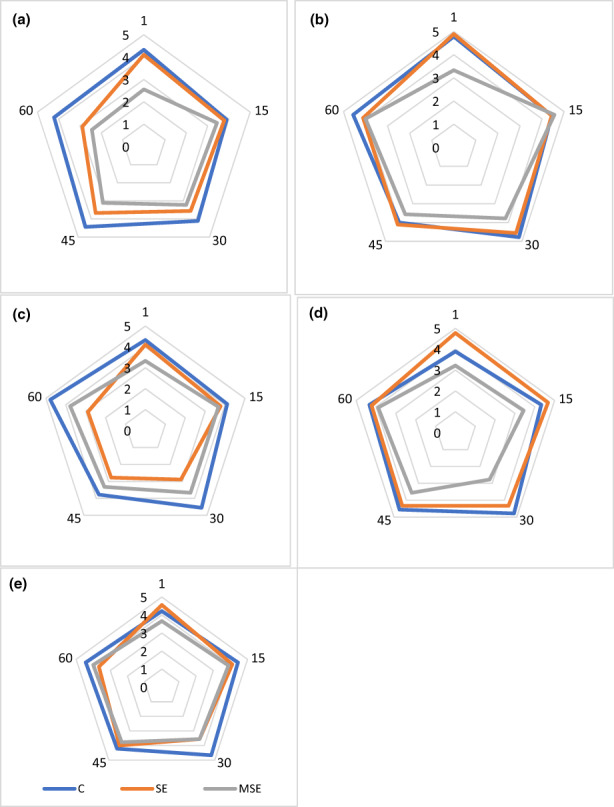
Sensory characteristics: flavor (a); odor (b); color (c); texture (d); overall acceptability (e) of microencapsulated seed extract (MSE); seed extract (SE) of the *Siah‐e‐Samarghandi* grape; and C = control cheese during the storage time.

The results of sensory properties including texture, taste, color, odor, and overall acceptability of the stored cheese at 4°C for 60 days are given in Figure 4a–e. Significant differences (*p* < .05) were observed for (flavor and color) parameters among C, SE, and MSE cheese samples at the end of storage time. In the same vein, Kim et al. (2017) found that beads containing chili pepper extract affect the flavor of fortified Gouda cheese. The result of this study is in contrast to previous studies wherein there have been reports of improved sensory properties of the extract‐enriched cheeses with micro‐coatings; for example, cheese containing grape seed powder and extract were significantly different in appearance, texture, and taste from the control sample; therefore, that increasing the level of grape seed powder in the cheese formulation has brought about high scores in the flavor of the product (Elgaml et al., 2018). They attributed the positive effect of using grape seed in cheese on the taste of the product to the presence of phenolic compounds. One of the reasons for this difference may be related to the kind of micro‐coating technique that affects odor and/or taste compounds (Elnaz & Saeed, 2020).

### 3.10 PV and AnV

As shown in Table 5, the PV and AnV values of all samples showed an increasing trend during the storage period for all samples. However, such an increase showed a slower tendency for the samples with SE and MSE compared with the control. For instance, the increased trend of C, SE, and MSE samples were 172.54%, 145.68%, and 118.75% for peroxide and 35.68%, 32.28%, and 17.24% for P‐anisidine values separately. So fat oxidation is reduced in fortified cheese. These results were supported by the nanoparticulation of olive leaf extract (OLE) encapsulated by nanoemulsions in soybean oil, in which the bioavailability, solubility, and antioxidant capacity of olive leaf extract (OLE) bioactive compounds increased compared to traditional dispersion methods (Mohammadi et al., 2016).

**TABLE 5:** Peroxide and para‐ansidine value in cheese samples during storage time.

Parameters	Cheese sample	Storage time	
		1	60
Peroxide value (milliequiv. of peroxide/1000 g sample)	C	0.51 ± 0.01^bB^	1.39 ± 0.01^aC^
	SE	0.81 ± 0.015^bC^	1.99 ± 0.005^aB^
	MSE	0.96 ± 0.01^bA^	2.1 ± 0.001^aA^
*P*‐anisidine	C	2.61 ± 0.005^bA^	3.54 ± 0.001aA
	SE	2.54 ± 0.005^bB^	3.36 ± 0.001^aB^
	MSE	2.32 ± 0.02^bC^	2.72 ± 0.01^aC^

*Note*: Microencapsulated seed extract (MSE), seed extract (SE) of the *Siah‐e‐Samarghandi* grape, and control cheese (C). All the results were the means ± standard deviations of triplicate determinations. Data within the different letters (A–C) in column and (a–b) in a row are significantly different (*p* ≤ .05).

### 3.11 Starter viability

**FIGURE 5:**
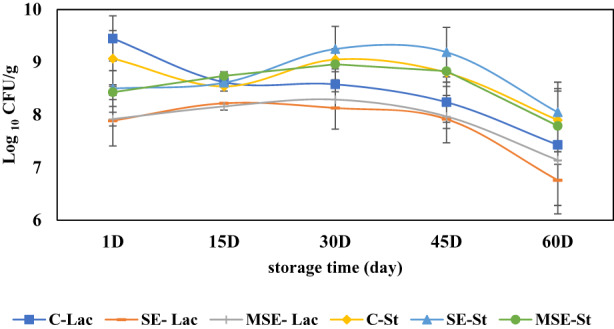
Microbial parameters of cheese samples supplemented with microencapsulated seed extract (MSE), seed extract (SE) of the *Siah‐e‐Samarghandi* grape, and control cheese (C) during the storage time. *Lactococcus lactis* (Lac) and *Streptococcus thermophilus* (St) count. Mean ± SD (*n* = 3).

The viability of *S. thermophilus* and *Lactococcus lactis* in SE, MSE, and C cheese samples during storage time is shown in Figure 5. Fortifying cheese with SE or MSE did not significantly change the survival rates of the cheese starter cultures containing *S. thermophilus* and *Lactococcus lactis* compared to the C. Decreased survival of cheese starter cultures followed similar trends in each sample (SE and MSE and C) during storage time, which suggests that protection with grape seed extract, directly or in the encapsulated form, did not influence the survival of both of the starter cultures. However, our results are in accord with earlier reports of grape seed extract fortified yogurt (Chouchouli et al., 2013; Yadav et al., 2018). Autolysis of starter bacteria is probably one of the major criteria to reduce all starters in cheese during the storage period. Moreover, the pH dropped during storage can greatly affect the growth of starters, as the optimum pH ranges for the growth of the starter were 6.5 and 6.3–6.9, for *S. thermophilus* and *L. lactis* subsp. *cremoris*, respectively (Borhanpour et al., 2021). Therefore, it seems that pH may adversely affect the survival of these bacteria in cheese during storage. In parallel, a reduction in the *Lactococcus* count of low‐fat cheddar cheese during storage was also reported (Akarca et al., 2016).

## 4 CONCLUSIONS

Most preservative components which are currently added to food as an additive are chemically produced or synthesized. The results of this study showed that the extract of *the Siah‐E‐Samarghandi* grape seed extract, as a natural additive, contains phenolic antioxidant compounds with moderate antioxidant activity. The addition of SE and MSE did not have any adverse effect on pH, acidity, and starter viable number, but decreased lipolysis and fat oxidation in cheese. Therefore, according to the results obtained in this study, the SE and MSE can be effectively used to produce functional UF‐Feta cheese with desirable and good antioxidant activity.

## AUTHOR CONTRIBUTIONS

**Saeed Sekhavatizadeh:** Methodology (lead); supervision (lead); writing – original draft (lead). **Nasim Abadariyan:** Software (equal). **Laya Ebrahimi:** Writing – review and editing (equal). **Mahboobeh Hasanzadeh:** Methodology (equal).

## CONFLICT OF INTEREST STATEMENT

The authors declare that they have no conflict of interest.

## ETHICAL APPROVAL

This study does not involve any human or animal testing.

## INFORMED CONSENT

Written informed consent was obtained from all study participants.

## Data Availability

The data are available from the corresponding author upon reasonable request.
